# Examining the lower range of the association between alcohol intake and risk of incident hospitalization with atrial fibrillation

**DOI:** 10.1016/j.ijcha.2020.100679

**Published:** 2020-11-29

**Authors:** Inger Ariansen, Eirik Degerud, Knut Gjesdal, Grethe S. Tell, Øyvind Næss

**Affiliations:** a Division of Mental and Physical Health, Norwegian Institute of Public Health, Oslo, Norway; b Department of Occupational Health Surveillance, National Institute of Occupational Health, Oslo, Norway; c Department of Cardiology, Oslo University Hospital Ullevål, Oslo, Norway; d Institute of Clinical Medicine, Oslo University, Oslo, Norway; e Department of Global Public Health and Primary Care, University of Bergen, Norway; f Department of Community Medicine and Global Health, Institute of Health and Society, Faculty of Medicine, University of Oslo, Oslo, Norway

**Keywords:** Alcohol, Atrial fibrillation, Health behavior

## Abstract

**Background:**

Evidence is sparse on the association between alcohol intakes in the lower range and risk of atrial fibrillation (AF). We aimed to investigate self-reported low and moderate alcohol intakes and subsequent risk of incident AF among current drinkers.

**Methods:**

Norwegian population-based health examination surveys assessing self-reported daily alcohol intake (mean grams per day) were linked to health and population registers. Hazard ratios (HR) (95% confidence interval) for time to incident (first) hospitalization with AF by alcohol intake level were assessed by Cox regression, with adjustment for educational level and cardiovascular risk factors except blood pressure.

**Results:**

The study population included 234,392 participants (49% men). Incident hospitalization with AF was identified in 5043 (2.2%) persons during a mean follow-up of 9 years. Compared to a very low alcohol intake of <1 unit weekly, a moderate consumption in the range of 1 to <2 units daily increased the risk of incident AF by 18% (HR 1.18 [1.06–1.32]). The average risk of incident AF increased by 9% per daily alcohol unit of 12 g (HR 1.09 [1.03, 1.14]). In sex-stratified analyses significant associations were found in men only.

**Conclusions:**

We found that less than two alcohol units/day significantly increased the risk of incident AF, however, in men only. Reduction of even a moderate alcohol intake may thus reduce the risk of AF at the population level.

## 1 Introduction

Atrial fibrillation (AF) is the most common arrhythmia in the general population, with a prevalence ranging from 0.7% in the age group 55–60 years to 17.8% in those aged 85 years and older [1]. AF contributes to a 3.5-fold increased risk of stroke and a 1.5–1.9-fold increased mortality [2]. Risk factors for AF include older age, male sex, concomitant cardiovascular conditions, diabetes, sleep apnea, family history of AF, and athletes’ endurance exercise level [3], as well as lifestyle factors including obesity, elevated blood pressure, smoking, and alcohol intake [4], [5].

High levels of alcohol consumption has long been suspected as a risk factor for cardiac arrhythmias after observations of what initially was referred to as the ‘holiday heart syndrome’ [6]. Several [7], [8], [9], [10], but not all [11], [12], [13], [14], [15], [16], [17] subsequent studies found an association between a high alcohol consumption and an increased risk of AF, and in some studies also among those reporting an intermediate intake [18]. Pooled estimates in meta-analyses have suggested that each additional unit of alcohol per day contributes to on average 8% increased risk of AF [18], [19]. The findings are generally more consistent in men than in women, and less robust in older populations [20]. Based on current evidence, the European Society of Cardiology’s guidelines on cardiovascular disease prevention recommend that alcohol intake should be limited to two alcohol units per day for men (20 g) and one unit per day for women (10 g) [21].

An important and unanswered question is whether the risk of AF increases even at lower alcohol intake levels of up to one or two units of alcohol per day. More recent findings and pooled estimates from prospective studies of AF risk indicate that low alcohol intake is not associated with increased risk of AF [22], [23]. However, the studies underlying the pooled estimates vary with regard to categorization of alcohol intake and choice of reference group. Many individual population-based studies have been statistically underpowered for study of the effect of low alcohol intake on AF risk, they have not distinguished between very-low and low alcohol intakes, and often included abstainers in the referent category, despite data showing that that there may be many “sick quitters” in this heterogeneous group [4], [18], [24], [25]. More data are thus required, as this could influence guidelines on cardiovascular disease prevention. If the risk of AF attributable to alcohol intake were primarily driven by high intake levels, the potential causal effect would involve a much lower share of the population than if the risk starts to increase even at lower levels. Given the established role of AF as risk factor for stroke and subsequent disability, it is important to further investigate this issue.

Analyzing 234,392 participants in Norwegian population-based cardiovascular health examination surveys, we aimed to study the risk of incident hospitalization with AF within low and moderate levels of alcohol intake, using very low-level drinking as the referent category. We also assessed whether the association varied by sex or educational level. As far as we know, this pooled dataset of Norwegian homogenous population-based cardiovascular health examination surveys represents the globally largest sample to study lower level alcohol intake and risk of AF, and its sample size is similar to the most recent international meta-analysis [23].

## 2 Methods

### 2.1 Data sources

The source population consisted of participants in Norwegian population-based health examination surveys; the Age 40 Program (1994–1999) invited all men and women in their early 40 s from 15 of Norway’s 19 counties, and the Cohort of Norway (CONOR, 1994–2001) included regional surveys in adult populations from eight counties, with average participation rates of 70% and 58%, respectively [26], [27]. These health survey data were linked to data from the National Registry, the National Education Database, and to a nationwide database of cardiovascular disease hospitalizations in the Cardiovascular Disease in Norway (CVDNOR) project [28], [29] using the personal identification number, unique to each Norwegian resident. The Regional Committee of Medical and Health Research Ethics in South East of Norway (11/1676) approved the study and gave exemption regarding written informed consent in the surveys where this was not obtained. The study protocol conforms to the ethical guidelines of the 1975 Declaration of Helsinki.

### 2.2 Design and study population

In this prospective cohort study we examined risk of incident hospitalization with AF by self-reported alcohol intake. The study population consisted of health survey participants reporting any amount of current drinking, who had complete data on alcohol intake and on the covariates adjusted for in the multivariable models, and who were at risk of incident AF.

### 2.3 Alcohol intake

Questions relevant to alcohol intake from the self-administered questionnaires differed between surveys. We used two approaches to estimate the average intake of alcohol in grams/day among current drinkers depending on the data available in each survey. The first approach used the question “How many glasses of beer, wine, or spirits do you usually drink during a two-week period”, which was answered separately for each beverage type (range 0–50, higher values truncated). For participants with data available on at least one beverage type, we estimated the average alcohol intake (grams/day) by converting glasses to grams of pure alcohol, adding up the grams, and dividing by 14. In the calculations we used the weight 789 g per liter pure alcohol and the following definitions: one glass of beer (33.3 cl, 4.5%, 11.8 g), one glass of wine (15 cl, 12%, 14 g), and one glass of hard liquor (4 cl, 40%, 12.6 g). The second approach combined drinking frequency obtained from the question “How often during the past 12 months have you consumed alcohol” with the reported number of glasses consumed per occasion from the question “When you drank alcohol, how many glasses did you usually drink”. The drinking frequency was reported in ordinal frequency categories, and converted into frequency per year as follows (“<1 time/month” = 6 times/year, “1 time/month” = 12 times/year, “2–3 times/month” = 30 times/year, “1 time/week” = 52 times/year, “2–3 times/week” = 130 times/year, and “4–7 times/week” = 286 times/year). The number of glasses per occasion was truncated to a maximum of 20. We multiplied the frequency per year with the number of glasses per occasion, divided this by the number of days in a year (365.25, and by assuming that 1 glass = 12.8 g of pure alcohol, we estimated the average intake of alcohol (grams/day).

### 2.4 Covariates

The National Education Database provided data on the highest attained education classified into either primary (up to 7 years in the 1960 s, up to 9 years from 1970 and later) representing compulsory primary and lower secondary school or lower levels, secondary (10–12 years) representing completion of first, second or third year of upper secondary school, or tertiary (13 years or longer) usually representing completion of college or a university degree, corresponding to the International Classification of Education 1997 categories 1–2, 3–4 and 5–8. Information on participants’ marital status (married, divorced, separated/widower, never married) at the time when the survey invitation letter was sent out, was available from the National Registry of Norway.

Information on smoking status (never, light former smoker, heavy former smoker, light current smoker, and heavy current smoker (light and heavy defined by <20 and ≥20 pack years, respectively)), level of physical activity (range 1 (sedentary) – 4 (highly active)), history of diabetes, history of cardiovascular disease (CVD) (angina pectoris, myocardial infarction or stroke), and family history of coronary heart disease (CHD), was obtained from self-administered questionnaires. Clinical measurements were performed by trained survey personnel, following standard protocols. The average of the second and third measurement of systolic blood pressure (mmHg) and heart rate (beats/min) from automatic oscillometric devices after two minutes of rest in a sitting position were used. Height and weight were measured to the nearest centimeter and half kilogram, respectively, and body mass index (BMI, kg/m^2^) was calculated. Serum concentrations in mmol/l of triglycerides, total cholesterol, and HDL-cholesterol were obtained from biochemical measurements in non-fasting blood samples.

### 2.5 Incident atrial fibrillation

The AF diagnosis was identified from national hospital inpatient admissions, retrieved from patient administrative systems in all hospitals during 1994–2009 in the CVDNOR project [28], [29]. Incident AF was defined as the first hospitalization with a main or secondary diagnosis of atrial fibrillation or atrial flutter (ICD-9:427.3, ICD-10: I48), with no previous hospitalization with AF prior to participation in the health survey or during a wash-out period of the first 7 years of the available hospitalization data (1994–2000). The length of this wash-out period was selected to minimize misclassification of recurrent cases as incident cases (7-year wash-out yields 11% misclassification [30]), while still allowing for a reasonably long study period. AF as secondary diagnosis represents an important source for identification of incident hospitalizations with AF. More than two thirds of all incident AF inpatient hospitalizations in Norway are identified from secondary diagnosis [30]. AF as secondary diagnosis was therefore included in our definition of incident hospitalization with AF.

The study population was followed from health survey participation to incident hospitalization with AF, or to censoring by death, emigration or end of follow-up 31st December 2009.

### 2.6 Statistical analyses

We present descriptive statistics according to categories of alcohol intake (“<2 g/day”, “2 to <12 g/day”, “12 to <24 g/day”, and “≥24 g/day”), and use analysis of variance and the Chi-Square test to test for differences. Cox Proportional Hazard modelling was used to estimate hazard ratios (HRs) and 95% confidence intervals (CIs) for the risk of incident AF. The proportionality assumption was checked by inspecting survival curves and Schoenfeld residuals against time. The models included alcohol intake, age, sex, smoking status, body mass index, diabetes, history of CVD, resting heart rate, physical activity, family history of CHD, serum cholesterol, serum triglycerides, educational level, and marital status. Alcohol was modelled both as a categorical variable with participants drinking “<2 g/day” as the reference category, and as a continuous variable (per 12 g increase). We performed stratified analyses by sex and by attained education (“primary”, “secondary”, and “tertiary”), and in addition tested for interaction by attained education to explore if the association between alcohol intake and risk of incident AF differed for higher educated relative to those with primary education only. For visualization of the functional relationship between alcohol intake and risk of incident AF, we fitted a model with alcohol intake as a continuous variable using penalized smoothed splines in Cox models adjusted for age and sex. The regression term (log HR) was plotted as a function of alcohol intake and the function centered at the mean value of the predictors. To aid interpretation of the sex-stratified effect estimates we performed a simplified post-hoc power analysis using chi square test comparing two independent proportions with a one-sided test and a significance level of 0.05.

## 3 Results

### 3.1 Study population

**Fig. 1:**
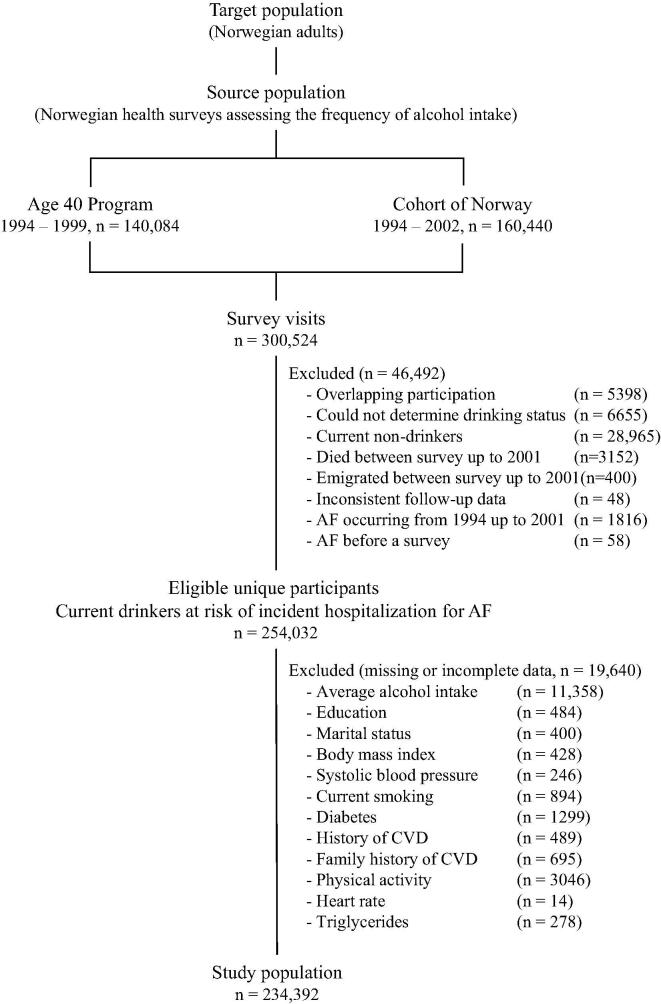
Flow-chart for defining the study population from Norwegian population-based health examination surveys, according to availability and completeness of data. Abbreviation: CVD, cardiovascular disease.

From the health examination surveys we selected one visit per individual, with priority to the visit with no missing values for alcohol drinking status, to the first visit for surveys with more than one wave, or to the visit from CONOR if a participant attended a survey both in CONOR and in the Age 40 Program. Current drinkers at risk of incident hospitalizations for AF and with complete data on covariates included in multivariable analyses, were considered eligible (n = 254,032). Of these, participants without data on alcohol intake quantity (n = 11,358) or covariates (n = 8,282) were excluded, resulting in a final study population of 234,392 participants. A flow chart of the selection process is provided (Fig. 1).

### 3.2 Baseline descriptive statistics

Men constituted 49% of the study population. The proportion of men increased with increasing alcohol intake, and was 76% and 85% in the upper categories drinking 12 to <24 g/day and ≥24 g/day, respectively. In both sexes’ alcohol intake was numerically positively associated with: having ever been married, average educational level, current smoking and serum HDL-cholesterol, and negatively associated with never having smoked (Table 1a, Table 1b). A J or U-shaped numerical relationship with alcohol intake category was observed for age, BMI, heart rate, systolic blood pressure, serum triglycerides, serum total cholesterol, diabetes, and history of CVD, suggesting that participants with an alcohol intake of “2 to <12 g/day” and “12 to <24 g/day” were younger, and had a more favorable cardiovascular risk profile in comparison with participants in the lowest and highest alcohol intake categories drinking “<2 g/day”, and “≥24 g/day”, respectively.

**Table 1a:** Descriptive statistics according to alcohol intake among 113,372 men in Norwegian health surveys.

	All men	Average intake of alcohol (grams per day)				
		<2 g/day	2 - <12 g/day	12 - <24 g/day	≥24 g/day	*p-value*
Participants	113,372 (1 0 0)	34,716 (31)	63,543 (56)	12,593 (11)	2520 (2)	
Alcohol, grams/day	6.0 ± 6.9	0.7 ± 0.7	5.8 ± 2.6	16.0 ± 3.1	35.1 ± 13.4	<0.001
Age, years	44 ± 10	46 ± 12	43 ± 9	43 ± 9	44 ± 10	<0.001
Ever married	26,562 (23)	7461 (22)	14,561 (23)	3655 (29)	885 (35)	<0.001
Educational level (1–8)	4.1 ± 1.6	3.8 ± 1.6	4.2 ± 1.6	4.4 ± 1.7	4.3 ± 1.7	<0.001
Primary education (1–2)	20,932 (19)	8211 (24)	10,260 (16)	1943 (15)	518 (21)	<0.001
Secondary education (3–4)	61,063 (54)	18,956 (55)	34,595 (54)	6359 (51)	1153 (46)	<0.001
Tertiary education (5–8)	31,377 (28)	7549 (22)	18,688 (29)	4291 (34)	849 (34)	<0.001
Current smoker	39,483 (35)	10,621 (31)	22,092 (35)	5481 (44)	1289 (51)	<0.001
Never smoker	41,466 (37)	14,059 (41)	23,286 (37)	3547 (28)	574 (23)	<0.001
Former light smoker	27,284 (24)	8442 (24)	15472 (24)	2876 (23)	494 (20)	<0.001
Former heavy smoker	5139 (4.5)	1594 (4.6)	2693 (4.2)	689 (5.5)	163 (6.5)	<0.001
Current light smoker	27,802 (25)	7522 (22)	16,104 (25)	3522 (28)	654 (26)	<0.001
Current heavy smoker	11,681 (10)	3099 (9)	5988 (9)	1959 (16)	635 (25)	<0.001
Physical activity (1–4)	2.2 ± 1.0	2.1 ± 1.0	2.2 ± 1.0	2.2 ± 1.0	2.1 ± 1.0	<0.001
BMI, kg/m^2^	26.3 ± 3.4	26.4 ± 3.6	26.2 ± 3.3	26.3 ± 3.3	26.3 ± 3.6	<0.001
Heart rate, bpm	71 ± 12	71 ± 12	70 ± 12	71 ± 12	73 ± 13	0.709
Systolic BP, mm Hg	134 ± 15	135 ± 16	133 ± 15	134 ± 15	136 ± 16	<0.001
Triglycerides, mmol/l	2.04 ± 1.36	2.07 ± 1.36	2.02 ± 1.32	2.07 ± 1.46	2.13 ± 1.64	0.759
Total cholesterol, mmol/l	5.77 ± 1.10	5.78 ± 1.11	5.76 ± 1.08	5.83 ± 1.15	5.85 ± 1.10	0.002
HDL–cholesterol, mmol/l	1.24 ± 0.33	1.19 ± 0.31	1.25 ± 0.32	1.30 ± 0.35	1.37 ± 0.40	<0.001
Diabetes	1642 (1.4)	760 (2.2)	728 (1.1)	115 (0.9)	39 (1.5)	<0.001
History of CVD	4190 (3.7)	1935 (5.6)	1851 (2.9)	310 (2.5)	94 (3.7)	<0.001
Family history of CHD	43,691 (39 )	13,608 (39)	24,445 (39)	4699 (37)	939 (37)	0.001

Abbreviations: BMI, body mass index; BP, blood pressure; HDL-cholesterol, high-density lipoprotein cholesterol; CVD, cardiovascular disease; CHD, Coronary heart disease. Values are presented as mean ± standard deviation and count (percentage). Group differences were assessed with analysis of variance and the chi-square test.

**Table 1b:** Descriptive statistics according to alcohol intake among 121,020 women in Norwegian health surveys.

	All women	Average intake of alcohol (grams per day)				
		<2 g/day	2 - <12 g/day	12 - <24 g/day	≥24 g/day	*p-value*
Participants	121,020 (1 0 0)	57,527 (48)	58,925 (49)	4,107 (3)	461 (0.4)	
Alcohol, grams/day	3.3 ± 4.2	0.5 ± 0.7	4.9 ± 2.4	15.6 ± 3.0	34.5 ± 12.3	<0.001
Age, years	43 ± 10	44 ± 11	42 ± 9	44 ± 9	45 ± 10	<0.001
Ever married	20,806 (17)	9648 (17)	10,237 (17)	807 (20)	114 (25)	<0.001
Educational level (1–8)	4.0 ± 1.6	3.8 ± 1.6	4.2 ± 1.7	4.6 ± 1.7	4.4 ± 1.8	<0.001
Primary education (1–2)	25,691 (21)	14,572 (25)	10,423 (18)	598 (15)	98 (21)	<0.001
Secondary education (3–4)	57,147 (47)	28,088 (49)	27,240 (46)	1648 (40)	171 (37)	<0.001
Tertiary education (5–8)	38,182 (33)	14,867 (26)	21,262 (36)	1861 (45)	192 (42)	<0.001
Current smoker	45,486 (38)	20,048 (35)	23,162 (39)	1997 (49)	279 (61)	<0.001
Never smoker	44,288 (37)	23,780 (41)	19,513 (33)	922 (22)	73 (16)	<0.001
Former light smoker	29,645 (25)	13,004 (23)	15,494 (26)	1059 (26)	88 (20)	<0.001
Former heavy smoker	1601 (1.3)	695 (1.2)	756 (1.3)	129 (3.1)	21 (4.6)	<0.001
Current light smoker	38,137 (32)	17,090 (30)	19,470 (33)	1420 (35)	157 (34)	<0.001
Current heavy smoker	7349 (6)	2958 (5)	3692 (6)	577 (14)	122 (27)	<0.001
Physical activity (1–4)	2.0 ± 0.9	1.9 ± 0.9	2.0 ± 0.9	2.1 ± 0.9	2.0 ± 0.9	<0.001
BMI, kg/m^2^	25.0 ± 4.1	25.5 ± 4.5	24.6 ± 3.7	24.3 ± 3.6	24.4 ± 3.9	<0.001
Heart rate, bpm	75 ± 12	76 ± 12	74 ± 12	74 ± 12	75 ± 12	<0.001
Systolic BP, mm Hg	125 ± 17	127 ± 18	124 ± 15.7	125 ± 16	127 ± 18	<0.001
Triglycerides, mmol/l	1.36 ± 0.84	1.44 ± 0.90	1.30 ± 0.77	1.30 ± 0.87	1.37 ± 1.06	<0.001
Total cholesterol, mmol/l	5.52 ± 1.09	5.62 ± 1.16	5.43 ± 1.03	5.47 ± 1.00	5.59 ± 1.15	<0.001
HDL–cholesterol, mmol/l	1.51 ± 0.37	1.46 ± 0.36	1.54 ± 0.38	1.65 ± 0.41	1.74 ± 0.48	<0.001
Diabetes	1248 (1.0)	794 (1.4)	434 (0.7)	17 (0.4)	3 (0.7)	<0.001
History of CVD	1862 (1.5)	1239 (2.2)	561 (1.0)	50 (1.2)	12 (2.6)	<0.001
Family history of CHD	49,808 (41)	24,263 (42)	23,765 (40)	1588 (39)	192 (42)	<0.001

Abbreviations: BMI, body mass index; BP, blood pressure; HDL-cholesterol, high-density lipoprotein cholesterol; CVD, cardiovascular disease; CHD, Coronary heart disease. Values are presented as mean ± standard deviation and count (percentage). Group differences were assessed with analysis of variance and the chi-square test.

### 3.3 Outcome

The number (%) of participants with an incident hospitalization with AF accumulated to 5043 (2.2%) during a mean of 8.7 (SD 1.2) years of follow-up. The number of participants with incident AF according to sex and alcohol intake, is presented in Table 2.

**Table 2:** Average alcohol intake and risk of incident atrial fibrillation among 234,392 participants in Norwegian health surveys.

		*Average alcohol intake (grams per day)*				
		*<2 g/day* *(n = 92,243)*	*2 - <12 g/day* *(n = 122,468)*	*12 - <24 g/day* *(n = 16,700)*	*≥24 g/day* *(n = 2,981)*	*Per 12 g/day increase* *(n = 234,392)*
**Total population at risk (n = 234,392)**						
Incident AF events, n (%)		2360 (2.6)	2196 (1.8)	390 (2.3)	97 (3.32)	5043 (2.2)
Hazard ratio (95% CI) of incident AF						
	Age and sex model	1.00	0.97 (0.91, 1.03)	1.10 (0.98, 1.22)	1.22 (1.00, 1.50)	1.06 (1.01, 1.11)
	Multivariable model	1.00	1.03 (0.97, 1.09)	1.18 (1.06, 1.32)	1.25 (1.02, 1.53)	1.09 (1.03, 1.14)

**Women at risk (n = 121,020)**						
Incident AF events, n (%)		1000 (1.7)	558 (0.9)	56 (1.4)	9 (1.9)	1623 (1.3)
Hazard ratio (95% CI) of incident AF						
	Age and sex model	1.00	0.92 (0.82, 1.02)	1.06 (0.81, 1.38)	1.22 (0.63, 2.36)	0.96 (0.84, 1.10)
	Multivariable model	1.00	0.98 (0.88, 1.09)	1.16 (0.88, 1.53)	1.34 (0.69, 2.59)	1.03 (0.90, 1.18)

**Men at risk (n = 113,372)**						
Incident AF events, n (%)		1360 (3.9)	1638 (2.6)	334 (2.7)	88 (3.5)	3420 (3.0)
Hazard ratio (95% CI) of incident AF						
	Age and sex model	1.00	1.00 (0.93, 1.07)	1.10 (0.97, 1.24)	1.23 (0.99, 1.53)	1.07 (1.01, 1.13)
	Multivariable model	1.00	1.03 (0.96, 1.11)	1.14 (1.01, 1.30)	1.23 (0.98, 1.53)	1.08 (1.02, 1.14)

Abbreviations: AF, atrial fibrillation; HR, hazard ratio; CI, confidence interval. HR with 95% CI are derived from Cox models. Average alcohol intake is presented as grams per day (g/day). The Age and sex model was adjusted for age and sex only. The multivariable model was adjusted for age, sex, education, marital status, smoking, physical activity, body mass index, resting heart rate, total cholesterol concentration, triglyceride concentration, diabetes, family history of coronary heart disease, and history of cardiovascular disease.

### 3.4 Average alcohol intake and risk of incident AF

**Fig. 2:**
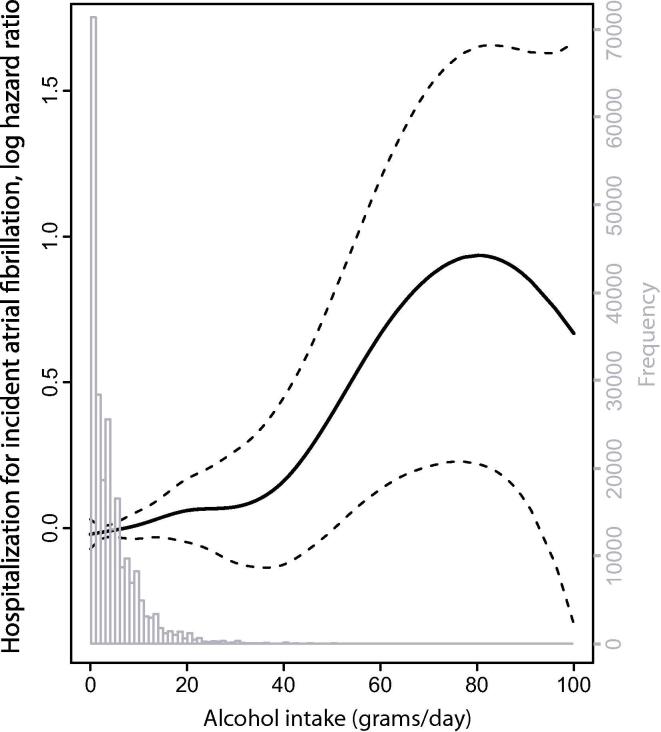
The distribution of alcohol intake among 234,392 Norwegian health survey participants and the relationship between alcohol intake and the risk of atrial fibrillation (5043 incident events) during a mean (SD) follow-up time of 8.7(1.2) years. Obtained using Cox proportional hazard model with age, sex, and alcohol intake fitted as a penalized smoothed spline. The solid line shows the spline and the stipulated lines depict twice the standard error (approximately a 95% CI). The distribution of the frequency of participants by alcohol intake is superimposed (in grey).

A visual presentation of the age and sex adjusted association (Fig. 2) between alcohol intake and the risk of incident AF indicates a non-linear relationship, characterized by a linear increase in risk at low to moderate intake levels and a steeper increase in risk at higher intake levels. Multivariable adjusted HRs and 95% CIs for the risk of incident AF according to a linear presentation of alcohol intake showed in average 9% increase in risk per unit (12 g) increase in alcohol intake. Participants drinking on average at least 1 unit daily, but less than 2 units daily (“12 to <24 g/day”), had 18% greater risk of incident AF compared to those drinking less than one unit weekly (“<2 g/day”), multivariable adjusted HR 1.18 (1.06, 1.32). For participants drinking 2 or more units per day, the corresponding risk was 25%. (Table 2). Overall, the multivariable adjustment resulted in a very modest increase in the age and sex adjusted HRs. The HRs did not change much when different cardiovascular risk factors, education or marital status were included individually to the age and sex adjusted model, and in supplementary analyses, neither did the potential mediator systolic blood pressure alter the HRs for alcohol substantially when included as a covariate (Supplementary Table S1).

### 3.5 Average alcohol intake and risk of incident AF according to sex and attained education

Stratified analyses of the association between alcohol intake and the risk of incident AF showed increased risk of AF by higher alcohol intake in men only (Table 2). The analyses showed HRs that were mostly in direction of a slight association between higher alcohol intake and risk of incident AF among women (Table 2) and among participants with both primary, secondary, and tertiary level of education (Table 3), but the confidence intervals were wide. The slope of the association did not differ substantially between men and women, or between the strata of attained education. In women, post-hoc power to detect significant differences for associations between each alcohol intake category (using very low intake (<2 g/day) as reference) and risk of incident AF was good for the low intake category (2 to <12 g/day) (100%), and poor for moderate (12 to <24 g/day) and higher (≥24 g/day) intakes (41% and 11%, respectively). In men, post-hoc power was good for the low and moderate intake categories (100% and 100%), and poor for the higher intake category (25%).

**Table 3:** Average alcohol intake and risk of incident atrial fibrillation in strata of attained education among 234,392 participants in Norwegian health surveys.

		*Average alcohol intake (grams/day)*					*Interaction*
HR (95% CI) of incident AF		*<2 g/day* *(n = 92,243)*	*2 - <12 g/day* *(n = 122,468)*	*12 - <24 g/day* *(n = 16,700)*	*≥24 g/day* *(n = 2,981)*	*Per 12 g/day* *(n = 234,392)*	Per 12 g/day * education HR (95% CI), p-value
Age and sex model							
	Primary education	1.00	1.02 (0.91, 1.13)	1.10 (0.85, 1.41)	1.18 (0.73, 1.91)	1.07 (0.97, 1.18)	Referent category
	Secondary education	1.00	0.97 (0.89, 1.06)	1.21 (1.04, 1.41)	1.25 (0.93, 1.69)	1.10 (1.03, 1.18)	1.06 (0.94, 1.19), p = 0.379
	Tertiary education	1.00	1.04 (0.90, 1.20)	1.09 (0.87, 1.35)	1.39 (0.98, 1.98)	1.07 (0.97, 1.18)	1.02 (0.89, 1.17) p = 0.786

Multivariable model							
	Primary education	1.00	1.07 (0.96, 1.19)	1.14 (0.88, 1.48)	1.15 (0.71, 1.88)	1.09 (0.98, 1.20)	Referent category
	Secondary education	1.00	0.99 (0.91, 1.08)	1.22 (1.05, 1.42)	1.21 (0.89, 1.63)	1.09 (1.02, 1.17)	1.04 (0.92, 1.17), p = 0.513
	Tertiary education	1.00	1.03 (0.89, 1.20)	1.07 (0.85, 1.33)	1.29 (0.90, 1.85)	1.04 (0.94, 1.16)	1.00 (0.87, 1.15), p = 0.987

Abbreviations: AF, atrial fibrillation; HR, hazard ratio; CI, confidence interval. Average alcohol intake is presented as grams per day (g/day). HR with 95% CI are derived from Cox models. The Age and sex model was adjusted for age and sex only. The multivariable model was adjusted for age, sex, marital status, smoking, physical activity, body mass index, resting heart rate, total cholesterol concentration, triglyceride concentration, diabetes, family history of coronary heart disease, and history of cardiovascular disease. The number of individuals with primary, secondary, and tertiary education was 46,632, 118,210, and 69,559, respectively. Interaction by attained education was tested to explore if the association between alcohol intake and risk of AF differed for higher educated relative to those with primary education. Test of interaction is presented as HR (95%CI) and p-values for the interaction term of average alcohol intake (per 12 g/day) and attained educational level (in three categories with primary education as reference category).

## 4 Discussion

In a large sample of participants in Norwegian health examination surveys, we observed that consumption of moderate (1–2 units per day) and higher (2 units or more) amounts of alcohol on average was associated with an 18% and 25% increased risk of incident hospitalization with AF relative to very low intakes of less than 1 unit per week, respectively. Intakes of less than 1 unit per day was not associated with an increased risk. These associations were observed in men only, and was somewhat strengthened by adjustment for cardiovascular risk factors and educational level, but did not differ substantially between groups defined by educational attainment. In women, the point estimates were in the same direction as for men, but the Cis were wide in line with the poorer power to detect differences in women. The risk of incident AF did not increase in a linear dose-dependent manner, but rather in a non-linear manner characterized by an initial linear increase in risk at lower intakes followed by a steeper increase in risk at intake levels exceeding 2 units per day.

### 4.1 Strengths and limitations

The main strength of this study is the large population-based sample that reduces the risk for random errors. Our study with individual-level data substantially adds to the current evidence base by having almost the same study-size as the largest recently published meta-analysis [23]. Participation rates were reasonably high; still, assuming that the heaviest drinkers were less likely to participate, non-responder bias may underestimate the effect of alcohol on AF. We were unable to identify participants who changed their alcohol intake before or after attending the survey. To reduce the risk of reverse causality concerning “sick quitters”, we excluded abstainers and used low-level drinking as the referent category [24]. We wanted to study the association of alcohol intake with the risk of incident AF, and in order to reduce the probability of including individuals with established AF, we excluded the first 7 years of hospitalizations with AF. This may have weakened the strength of our estimates, as true incident AF cases might have been excluded. However, it increases the internal validity of the study by reducing bias from the misclassification of “sick quitters” into the low-drinking reference group.

In a previous study from the same source population, we evaluated the accuracy of self–reported drinking frequency by the use of HDL–cholesterol concentrations as a biomarker [31]. Here, we found a dose-response association of self-reported drinking frequency with increasing HDL–cholesterol levels, and an estimated underreporting of over 50%. Since the estimated underreporting seemed to be systematic across alcohol intake categories, this misclassification bias would not markedly affect the strength of the association, but would, however, limit the generalizability of our findings. One self-reported drink/day may in fact represent two drinks/day, as suggested by the HDL-cholesterol level.

In the present study the definition of AF cases was based on registered diagnose codes from inpatient hospitalization, retrieved from hospital administrative systems. AF diagnoses from inpatient hospitalizations are usually based on the current ECG, but may be based on a prior AF diagnose described in available reports i.e. from outpatient clinics, general practitioners, or from prescription medication indication, especially if AF is a secondary diagnosis. We did not have access to hospital medical records or results of ECGs, and could not verify the AF diagnoses in our study population. In another Norwegian cohort, however, 74% of the AF hospital discharge diagnoses were confirmed by ECG and medical records from hospitals and general practitioners [32]. Corresponding Scandinavian registries have published high positive predictive values; an AF diagnosis in the Swedish Patient Registry was confirmed in 97% of a random sample [33], and in the Danish Patient Registry an AF diagnosis was confirmed in 93% in a population-based cohort [34], whereas a review suggested a sensitivity of AF hospital diagnoses of 79% [35]. Our study did not include AF patients who were diagnosed and treated only in primary care or outpatient clinics, which may comprise up to 22% of the AF population [30], [36]. However, a substantial proportion of AF patients can be identified from incident inpatient hospitalization, of which AF as secondary diagnosis constitute over two thirds of the cases [30]. In our study, incident AF cases were thus identified from both main and secondary hospital discharge diagnoses, and our results may be generalized to patients with AF episodes that require inpatient hospitalization (such as cardioversion in persistent AF or acute need of frequency control), or to AF patients with another condition requiring hospitalization.

The true date of the first time AF was diagnosed may have been prior to the date of hospitalization, as AF may have been diagnosed earlier i.e. in general practice or at an outpatient clinic. We would expect that this was the case for a large proportion of the AF patients hospitalized with AF as their secondary diagnosis. AF as secondary diagnosis may, however, be discovered and diagnosed for the first time at hospitalization for another main cause. Finally, the available hospitalization data restricted our time horizon of follow-up to 2009.

### 4.2 Interpretation

Previous observational findings of AF risk by alcohol consumption within low to moderate intakes have been summarized in a meta-analysis with 249,496 participants from nine studies [23]. That analysis showed that low alcohol intake of up to one unit of alcohol per day was not associated with risk of incident AF, and that moderate intake yielded increased risk of incident AF in men only. Similar conclusions were drawn from analyses involving 47,002 participants in the third wave of the Norwegian Nord-Trøndelag Health study (HUNT 3) that suggested a safe limit for AF risk at an alcohol intake of less than 1 drink per day in women and 2 drinks per day in men, in the absence of risky drinking [22].In line with the meta-analysis [23], our findings in 234,392 participants suggest that there is an increased risk of incident AF within the range from one up to two units per day. The findings were consistent in strata of men only, and although the risk estimates were in the same direction among women, the much lower power to detect differences among women prevent a clear conclusion for the association among women. Also, the risk of incident AF by higher alcohol intake was consistent and similar across all educational groups. This is in contrast to our findings for cardiovascular mortality, studied previously in a partly overlapping population. In that study, we showed that the mortality risk of cardiovascular disease according to alcohol intake was less favorable among the socioeconomically disadvantaged, and correlated strongly with educational level [31].

Potential pathophysiological mechanisms by which alcohol increases risk of AF include a direct toxic effect [37], short term electrophysiological effects related to binge drinking and withdrawal [38], and long term effects through electrical and structural remodeling [39], elevated blood pressure [40], [41], and dilatation of the atria [42], the latter effect is restricted to habitual intake, and not to binge drinking [43]. Adjustment for potential other confounders, including the available markers of autonomic activation; heart rate and blood pressure, did not materially influence our results. The consistency in effect estimates across sex and educational groups give credence to the hypothesis of a potential causal association between alcohol consumption and incident AF. If this relationship is causal, limiting moderate alcohol intake in the general population may have more pronounced implications for prevention of AF and subsequent stroke than if the risk of AF is limited to high consumption only.

## 5 Conclusion

Results from the present study suggest that even moderate alcohol intake is associated with increased risk of incident AF. The findings were consistent in strata of men only, although the risk estimates were in the same direction among women. The recognition of alcohol consumption as a modifiable risk factor for AF in men, even at moderate intake levels, has important implications for public health, given the widespread use of alcohol and the importance of AF as a risk factor for subsequent stroke.

## Declaration of Competing Interest

The authors declare that they have no known competing financial interests or personal relationships that could have appeared to influence the work reported in this paper.
