# Supplementary material for: Examining the lower range of the association between alcohol intake and risk of incident hospitalization with atrial fibrillation
Source: Int J Cardiol Heart Vasc. 2020 Nov 29;31:100679. doi: 10.1016/j.ijcha.2020.100679 (PMC8164130; doi:10.1016/j.ijcha.2020.100679)
Supplement: Supplementary data 1 [file mmc1.docx]

## Supplementary Table S1. Covariate influence on the age and sex-adjusted association between alcohol intake and risk of incident atrial fibrillation among 234,932 participants in Norwegian health surveys.

|  | ***Average alcohol intake (grams per day)*** | | | |
| --- | --- | --- | --- | --- |
| HR (95 % CI) of incident AF | ***<2 g/day (n = 92,243)*** | ***2 - <12 g/day (n = 122,468)*** | ***12 - <24 g/day***  ***(n =16,700)*** | ***≥24 g/day (n = 2981)*** |
| Crude | 1.00 | 0.69 (0.65, 0.73) | 0.91 (0.81, 1.01) | 1.30 (1.06, 1.59) |
| Age | 1.00 | 1.08 (1.02, 1.15) | 1.40 (1.26, 1.56) | 1.66 (1.35, 2.03) |
| **Age + sex** | **1.00** | **0.97 (0.91, 1.03)** | **1.10 (0.98, 1.22)** | **1.22 (1.00, 1.50)** |
|  |  |  |  |  |
| Age + sex + smoke | 1.00 | 0.95 (0.90, 1.01) | 1.06 (0.95, 1.18) | 1.15 (0.94, 1.42) |
| Age + sex + physical activity | 1.00 | 0.98 (0.93, 1.04) | 1.12 (1.01, 1.25) | 1.24 (1.01, 1.53) |
| Age + sex + BMI | 1.00 | 0.99 (0.94, 1.06) | 1.13 (1.02, 1.27) | 1.24 (1.01, 1.52) |
| Age + sex + heart rate | 1.00 | 0.97 (0.91, 1.03) | 1.10 (0.98, 1.22) | 1.23 (1.00, 1.51) |
| Age + sex + serum triglycerides | 1.00 | 0.97 (0.91, 1.03) | 1.10 (0.99, 1.23) | 1.23 (1.00, 1.50) |
| Age + sex + serum cholesterol | 1.00 | 0.97 (0.91, 1.03) | 1.10 (0.98, 1.22) | 1.22 (1.00, 1.50) |
| Age + sex + diabetes | 1.00 | 0.97 (0.92, 1.03) | 1.11 (0.99, 1.23) | 1.22 (1.00, 1.50) |
| Age + sex + history of CVD | 1.00 | 0.98 (0.93, 1.05) | 1.13 (1.01, 1.26) | 1.25 (1.02, 1.54) |
| Age + sex + family history of CHD | 1.00 | 0.97 (0.91, 1.03) | 1.11 (0.99, 1.23) | 1.23 (1.01, 1.51) |
| Age + sex + attained education | 1.00 | 1.00 (0.94, 1.06) | 1.16 (1.04, 1.29) | 1.29 (1.05, 1.58) |
| Age + sex + marital status | 1.00 | 0.97 (0.91, 1.03) | 1.09 (0.97, 1.21) | 1.20 (0.98, 1.47) |
| Age + sex + systolic blood pressure | 1.00 | 0.98 (0.92, 1.04) | 1.10 (0.99, 1.23) | 1.22 (0.99, 1.49) |

HR and 95% CI derived from Cox models. Abbreviations: AF; atrial fibrillation; HR, hazard ratio; CI, confidence interval; BMI, body mass index; CVD; cardiovascular disease, CHD; Coronary heart disease.
